# Genomic architecture of antimicrobial resistance and mobile genetic elements in *Salmonella enterica* isolated from necropsied cattle in central Kentucky

**DOI:** 10.1128/spectrum.01581-26

**Published:** 2026-08-31

**Authors:** Nada A. Fahmy, Fayza Kouadri, Logan Burns, Beth Johnson, Vaneet Arora, Yosra A. Helmy

**Affiliations:** 1 Department of Veterinary Science, Martin-Gatton College of Agriculture, Food, and Environment, University of Kentucky 4530, Lexington, Kentucky, USA; 2 Division of Lab Services, Kentucky Department for Public Health 98263, Frankfort, Kentucky, USA; 3 Department of Pathology and Laboratory Medicine, College of Medicine, University of Kentucky 4530, Lexington, Kentucky, USA; Newcastle University, Newcastle Upon Tyne, United Kingdom

**Keywords:** antimicrobial resistance, mobile genetic elements, non-typhoidal *Salmonella *, One Health, whole-genome sequencing

## Abstract

Non-typhoidal *Salmonella* remains a leading global cause of foodborne disease and an important contributor to the global burden of antimicrobial resistance (AMR) within the One Health framework. Cattle serve as an important reservoir, facilitating the dissemination of antimicrobial-resistant *Salmonella* through the food chain and the environment. In this study, we analyzed the genomes of 27 *Salmonella enterica* isolates recovered from necropsied cattle in central Kentucky to characterize their genomic AMR, virulence, and mobile genetic element profiles. Multilocus sequence typing (MLST) identified 12 sequence types (STs), with ST10 (*S*. Dublin) representing the dominant lineage (25.9%). Genomic screening revealed 4,618 virulence gene occurrences, including conserved factors associated with secretion and adhesion, as well as variable toxin and immune evasion genes. Antimicrobial resistance genes (ARGs) were detected across the isolate collection, with resistance primarily associated with efflux systems (62.1%) and genes conferring resistance to aminoglycosides, tetracyclines, sulfonamides, phenicols, and β-lactams. Plasmids were detected in 77.8% of isolates, integrons were identified in 11.1% of isolates, and prophages and insertion sequence (IS) elements were widely distributed. While most resistance loci were chromosomal (63.5%), plasmid-associated ARGs (11.7%) demonstrated significantly greater proximity to IS elements, suggesting an increased potential for mobilization. Pan-genome analysis revealed an open pan-genome structure with a limited core (19.9%) and a dominant accessory genome (77.6%). Core-genome phylogenetic analysis incorporating 97 publicly available genomes demonstrated close clustering of cattle-associated isolates with isolates from food, human, and environmental sources, suggesting the presence of shared or closely related lineages across interconnected One Health compartments. These findings characterize the genomic architecture of AMR in cattle-associated *Salmonella* and highlight the potential contribution of mobile genetic elements to shaping AMR evolution and dissemination across interconnected One Health systems.

**IMPORTANCE:**

This study reveals that multidrug-resistant *Salmonella enterica* in cattle is sustained by a dual genomic architecture in which resistance determinants are both chromosomally embedded and mobilized through plasmids, prophages, and insertion sequence (IS) elements. The presence of plasmid-borne resistance genes and IS elements suggests their potential involvement in transposition-mediated dissemination. The predominance of chromosomal resistance may indicate greater persistence of resistance determinants within bacterial lineages. The dominance of the ST10 (*S.* Dublin) lineage and its close genomic relatedness to food-associated isolates, together with an open pan-genome structure, suggest ongoing circulation and genomic diversification of high-risk lineages across interconnected animal, food, and environmental systems.

## INTRODUCTION

*Salmonella enterica (S. enterica*), particularly non-typhoidal *Salmonella* (NTS), is transmitted to humans and animals primarily through the consumption of contaminated food and water (1). It remains a major global public health concern, resulting in approximately 1.35 million illnesses and 420 deaths annually and imposing an estimated economic burden of approximately $17.1 billion each year in the United States alone (2, 3). Despite intensive regulatory efforts and reductions in NTS burden associated with poultry and beef commodities, rates of human salmonellosis in the United States have remained largely unchanged over the last few years (4, 5). In cattle, *Salmonella* is an important pathogen associated with acute enterocolitis, septicemia, and epizootic abortion (6). The pathogen can also persist within peripheral lymph nodes, where it may evade standard carcass surface interventions. When contaminated lymph nodes are incorporated into ground beef, *Salmonella* may be introduced directly into the food supply (7). This risk is amplified in feedlot environments, where repeated cycles of bacterial shedding, environmental contamination, and re-exposure sustain persistent reservoirs of infection (8). These transmission cycles facilitate subclinical colonization, allowing clinically healthy cattle to harbor and shed the pathogen without being detected (9). Consequently, multidrug-resistant (MDR) strains may enter the food chain despite standard mitigation procedures, complicating source attribution during outbreaks (10). The increasing prevalence of MDR NTS is particularly concerning because it can shift disease outcomes from self-limiting gastroenteritis toward invasive infections that are more difficult to treat (11).

The emergence and persistence of the MDR phenotypes are supported by a highly dynamic genomic architecture of *Salmonella* (12). This genomic plasticity enables NTS to rapidly adapt to antimicrobial pressure through chromosomal mutations, regulatory changes affecting efflux systems, and the acquisition of mobile genetic elements (MGEs) (13). Although chromosomal mutations and efflux regulation can contribute to clinically significant resistance and treatment failure, the rapid dissemination of MDR is primarily driven by the mobilome, including plasmids, transposons, integrons, insertion sequence (IS) elements, and bacteriophages. Plasmids are frequently implicated in MDR *Salmonella* and represent an important reservoir of antimicrobial resistance genes (ARGs). The IS elements can further accelerate the evolution of resistance by mediating replicative transposition, plasmid rearrangements, and fusion events, thereby reshaping resistance regions and promoting the emergence of successful MDR genomic architectures (14–16). In a mobilome-focused analysis of 1,817 complete *Salmonella* genomes collected from 58 countries, plasmids harbored approximately 69.8% of identified ARGs, while specific prophages contributed to plasmid plasticity and ARG acquisition (17).

Addressing this threat requires a One Health approach that recognizes cattle as an important reservoir at the human–animal–food interface (18). However, conventional low-resolution typing methods have limited ability to distinguish closely related isolates and accurately trace *Salmonella* transmission across cattle reservoirs, food products, and human populations (19, 20). High-resolution genomic analyses are therefore needed to characterize resistance determinants, resolve transmission relationships, and track the dissemination of MDR strains across these interconnected settings (21). Whole-genome sequencing (WGS) has emerged as a transformative tool for surveillance and characterization of foodborne pathogens (18). It provides comprehensive genomic information for strain subtyping, antimicrobial resistance (AMR) and virulence profiling, mobile genetic element (MGE) analysis, and phylogenetic reconstruction, thereby improving source attribution and transmission tracking (22). Despite the growing availability of global genomic datasets, high-resolution regional studies integrating the resistance architecture and transmission dynamics of cattle-associated *Salmonella* remain limited.

In our previous study, we identified 27 MDR isolates from necropsied cattle submitted to the University of Kentucky Veterinary Diagnostic Laboratory (UKVDL) during 2022–2023. These isolates exhibited extensive antimicrobial resistance, biofilm-forming ability, and virulence-associated characteristics; however, the genomic mechanisms underlying these phenotypes remain unresolved (23). In the present study, we applied WGS to characterize the genomic architecture of AMR and virulence in these isolates, with a particular emphasis on the distribution of resistance determinants across chromosomal and MGEs. We further examined the mobilome, pan-genome structure, and phylogenetic relationships of the isolates in comparison with publicly available genomes. This integrated genomic approach provides insights into the persistence, evolution, and potential dissemination of MDR *Salmonella* across the cattle–food–human interface.

## MATERIALS AND METHODS

### Whole-genome sequencing and genome assembly

The 27 MDR *S. enterica* isolates analyzed in this study were selected from a previously described collection obtained from cattle necropsy cases submitted to the UKVDL between 2022 and 2023 (23). Isolates were revived from cryopreserved stocks on blood agar (Hardy Diagnostics, CA, USA) and incubated at 37°C for 24 h (24, 25). DNA extraction was performed using the DNeasy Blood & Tissue Kit (Qiagen, MD, USA) following the manufacturer’s instructions for downstream applications. Genomic DNA concentration and purity were initially assessed using a NanoDrop spectrophotometer (Thermo Fisher Scientific, MA, USA) (14, 24). Genomic DNA libraries were prepared using the Illumina DNA Prep Kit (Illumina, CA, USA), and sequencing was performed on the Illumina MiSeq platform with 2 × 150 bp paired-end reads. Adapter sequences were removed from paired-end reads using Trimmomatic (v0.40) (26). *De novo* assembly was performed with SPAdes (v4.0) (27), and raw read quality assessment was performed using FastQC (v0.12.1) (https://github.com/s-andrews/fastqc) (28). Raw sequence data for the assembled contigs were annotated using Prokka and PGAP (v6.7) (NCBI Prokaryotic Genome Annotation Pipeline) and are available in the NCBI GenBank assembly database (29) under BioProjects PRJNA1086499 and PRJNA1237852; accession numbers are available in Table S1. *In silico* serotype predictions were confirmed using SeqSero2 (https://github.com/denglab/SeqSero2) (30). Multilocus sequence typing (MLST) was performed based on seven housekeeping gene loci, comprising *aroC, dnaN, hemD, hisD, purE, sucA,* and *thrA*. Genome assemblies were analyzed using the MLST tool (https://github.com/tseemann/mlst), which assigns allelic profiles based on the *S. enterica* MLST scheme curated by the PubMLST database (31). A minimum spanning tree (MST) was generated using PHYLOViZ based on allelic differences across the MLST loci (32).

### *In silico* analysis of virulence and antimicrobial resistance genes (ARGs)

ARGs were identified from assembled genome contigs by matching to the Comprehensive Antibiotic Resistance Database (CARD) gene identifiers, and the matches were retained for downstream analysis. The ARGs were classified using the CARD ontology, which includes resistance mechanisms (33). Only high-confidence hits (perfect and strict) were included in the downstream analysis. Virulence-associated genes were identified with ABRicate using the Virulence Factor Database (VFDB) (https://github.com/tseemann/abricate). Virulence genes were categorized based on VFDB functional classifications (https://www.mgc.ac.cn/VFs/main.htm). For both databases, hits were filtered using thresholds of ≥80% nucleotide sequence identity and ≥80% sequence coverage.

### Characterization of plasmids and mobile genetic elements (MGEs)

Mobile genetic elements were identified from draft genome assemblies using complementary bioinformatic tools targeting plasmids, IS elements, and integron-associated regions. Plasmid content and mobility predictions were assessed using the MOB-suite (https://github.com/phac-nml/mob-suite). Genome assemblies were analyzed using default MOB-suite parameters to infer plasmid presence and predicted mobility (34). Putative prophage regions were detected using PHIGARO (https://github.com/bobeobibo/phigaro) (35). For IS detection, we used ISEScan (https://github.com/xiezhq/ISEScan) (36). Integron-associated sequences were screened using IntegronFinder (https://github.com/gem-pasteur/Integron_Finder) to identify integrases and associated gene cassette arrays (37). ARGs were identified using CARD and subsequently integrated with plasmid and prophage predictions to determine their genomic context. ARGs were classified based on direct genomic coordinate overlap as chromosomal, plasmid-borne, or prophage-associated. For each ARG locus, the genomic distance to the nearest IS element was calculated. The ARG loci were classified as IS-proximal when an IS element occurred within ≤5 kb or ≤10 kb of the ARG locus (38). Chromosomal ARG-containing contigs were extracted and screened for similarity to known *Salmonella* genomic islands (SGIs) using BLASTN against SGI1 and SGI2 reference sequences. BLASTN hits were assessed based on percent identity, alignment length, and query coverage. Low-coverage alignments were not considered evidence of complete SGI-like resistance islands (39).

### Pan-genome analysis

A comparative pan-genome analysis was conducted to characterize the conserved and accessory genomic repertoires of 27 *S. enterica* isolates. To provide a broader comparative genomic context, an additional set of 97 publicly available *S. enterica* genomes was retrieved from the NCBI Pathogen Detection database (https://www.ncbi.nlm.nih.gov/pathogens/) based on the serotype and sequence type found in our study (File S1). Genomes were selected to represent a wide range of serotypes, STs, and host sources, including animal, food, environmental, and human-associated isolates, thereby enabling comprehensive comparative analyses (40). All genome assemblies were annotated using Prokka (v1.14.6) (41). Pan-genome analysis was conducted using Roary (v3.13.0), which clusters homologous protein sequences into orthologous gene groups based on BLASTp comparisons. Gene clustering was performed using a 95% amino acid sequence identity threshold, which is widely used in bacterial pan-genome studies. Gene clusters were categorized according to their prevalence across the data set as follows: core genes (present in ≥99% of isolates), soft-core genes (≥95%–<99%), shell genes (≥15%–<95%), and cloud genes (<15%) (42).

### Core genome phylogenetic analysis

The core genes shared across all genomes were identified using Roary (v3.13.0) for pan-genome analysis, with standardized genome annotations described above. Concatenated core gene sequences were used to generate a multiple sequence alignment. Phylogenetic reconstruction was performed based on a core-gene alignment. Evolutionary distances were calculated using the Jukes–Cantor substitution model (43, 44). A phylogenetic tree was constructed using the neighbor-joining method (45). The reliability of the tree topology was assessed via bootstrap analysis with 1,000 replicates (46). The resulting phylogenetic tree was visualized and annotated in Interactive Tree of Life (iTOL) for downstream comparative analyses (https://itol.embl.de/) (47).

### Statistical analysis

Differences in ARG distribution across genomic compartments were evaluated using two-sided Fisher’s exact tests. Odds ratios (ORs) with 95% confidence intervals (CIs) were calculated to quantify enrichment. *P* values were adjusted using the Benjamini–Hochberg false discovery rate (FDR) method. Associations between total ARG burden and isolate-level MGE features were assessed using Spearman’s rank correlation. All statistical analyses were conducted in R (v3.6.0), and FDR-adjusted *P* < 0.05 was considered statistically significant. Graphical summaries, including bar plots, heatmaps, and correlation plots, were generated in R (v3.6.0).

## RESULTS

### Genomic features, serovar, and sequence type (ST) of *Salmonella* isolates

**Fig 1:**
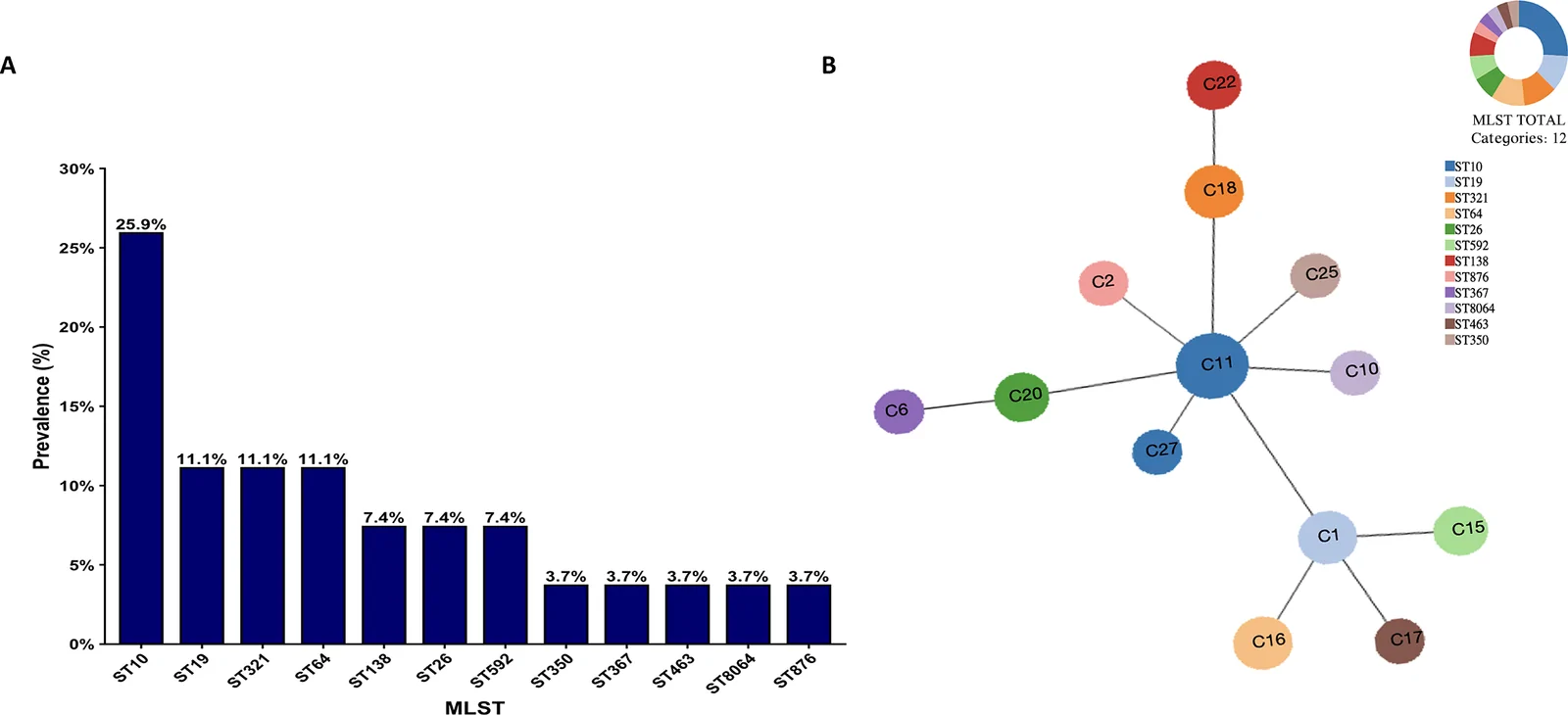
Distribution and allelic relatedness of sequence types among 27 *S. enterica* isolates. (**A**) Distribution of sequence types (STs). ST10 was the most frequent ST, followed by ST19, ST321, and ST64, whereas ST350, ST367, ST463, ST8064, and ST876 were each represented by a single isolate. (**B**) Minimum spanning tree illustrating the genetic relationships among the identified STs based on the allelic profiles of seven housekeeping genes (*aroC, dnaN, hemD, hisD, purE, sucA,* and *thrA*). Each node represents a distinct ST, and node size is proportional to the number of isolates assigned to that ST. Edges indicate allelic differences between STs, with shorter distances representing closer genetic relatedness.

*De novo* genome assembly produced draft genomes with an average sequencing depth of approximately 58×, providing sufficient coverage for comprehensive genomic characterization. The resulting assemblies consisted of 26–47 contigs per genome, with total assembly sizes ranging from 4.61 to 5.4 Mb, consistent with the expected genome size of *S*. *enterica*. Assembly continuity was supported by N50 values ranging from 158,500 to 537,500 bp; only one isolate had an N50 of 4,842 bp. The GC content was highly conserved across isolates, with a mean of approximately 52.1%. *In silico* serotyping of the 27 *S. enterica* isolates identified 12 distinct serotypes, with *S*. Dublin being the most prevalent serotype (25.9%; 7/27), followed by *S*. Typhimurium and *S*. Muenster (11.1%; 3/27 each). A discrepancy was observed for isolate C21, which was identified as *S.* Anatum by phenotypic serotyping but as *S.* Dublin by genomic serotyping. The remaining serotypes were each represented by ≤2 isolates. The MLST analysis identified 12 distinct STs among the 27 *Salmonella* isolates. ST10, corresponding to S. Dublin, was the most prevalent lineage, accounting for 25.9% (7/27) of the isolates. This was followed by ST19, ST321, and ST64, each representing 11.1% (3/27) of the isolates. Additional sequence types, including ST26, ST592, and ST138, were detected at lower frequencies (7.4%, 2/27 each), while the remaining STs (ST876, ST367, ST8064, ST463, and ST350) were each represented by a single isolate (3.7%, 1/27 each) (Fig. 1A and B; Table S1).

### Virulence gene profile

**Fig 2:**
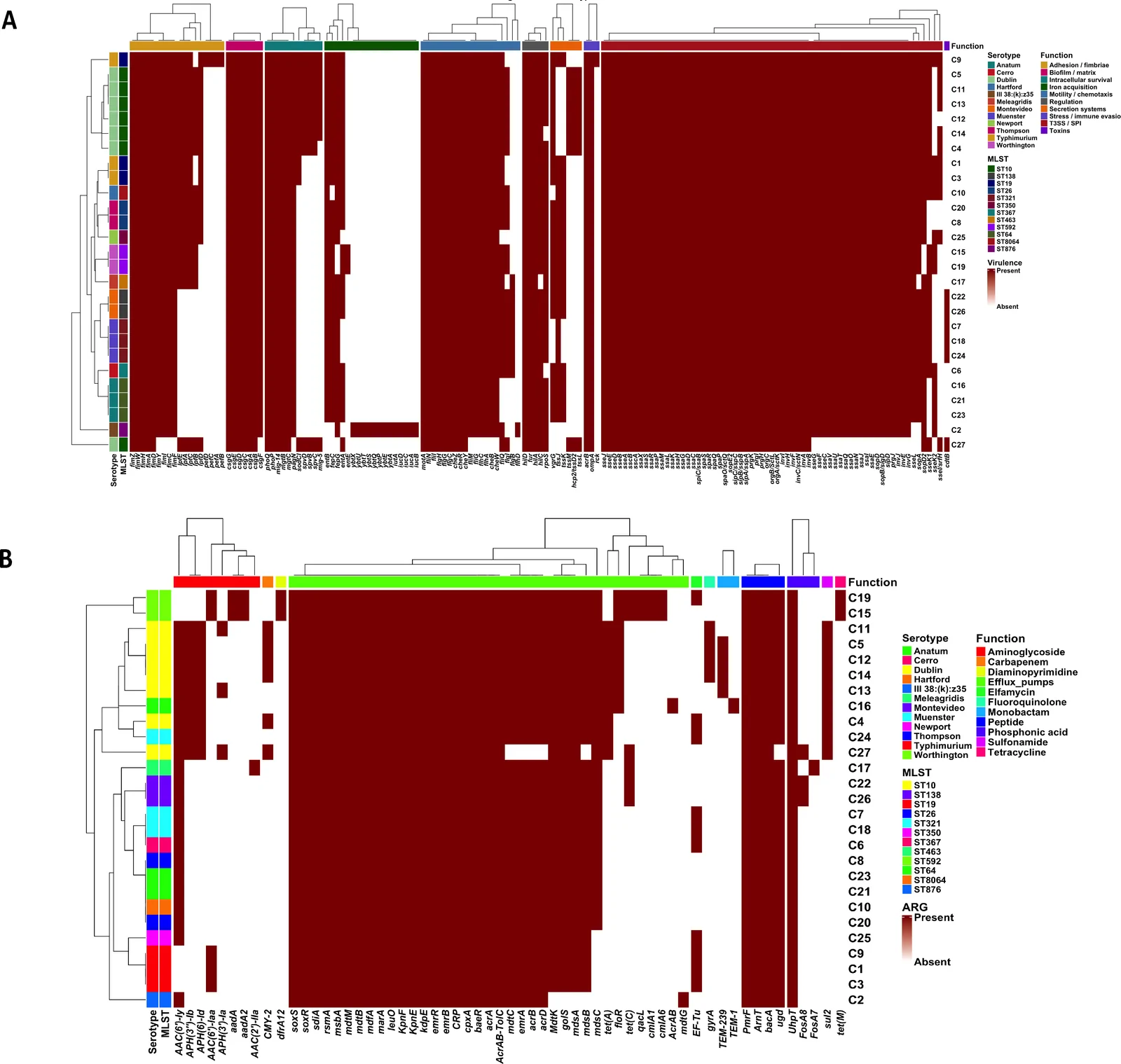
Distribution of virulence-associated genes and antimicrobial resistance genes among 27 *S. enterica* isolates. (**A**) Heatmap showing the distribution of virulence-associated genes across the isolates. (**B**) Heatmap showing the distribution of antimicrobial resistance genes (ARGs). Rows represent individual isolates, and columns represent genes. Hierarchical clustering of isolates and genes was performed to identify similarities in virulence and ARG profiles. The top annotation bars indicate the predicted functional categories of the genes, whereas the left-side annotations indicate isolate serotype and multilocus sequence type (MLST).

Virulence factor-associated gene profiling identified a total of 4,618 genes involved in pathogenicity and host interactions across 27 *S. enterica* isolates. Each isolate harbored more than 100 virulence-associated genes, indicating a broadly conserved virulence repertoire. Type III secretion system (T3SS) and *Salmonella* pathogenicity islands (SPIs) encoding genes were highly conserved across isolates. Specifically, the invasion protein A-encoding gene, *invA*, and the invasion protein B-encoding gene, *sipB,* and intracellular survival-associated genes (*steC* and *steD*) were present in 100% of the isolates. Additionally, genes associated with adhesion (*fimA*, *fimD, fimH, fimW,* and *fimZ*), biofilm formation (*csgA*, *csgB, csgC,* and *csgD*), and motility (*flgH, flgC,* and *motA*) were also present at 100%, supporting their roles in colonization and persistence. In contrast, iron acquisition genes exhibited greater variability among isolates. For example, the gene related to enterobactin biosynthesis protein (*entB*) was the most prevalent (92.6%), followed by the ferrienterobactin transport ATP-binding protein gene b*fepC* (88.9%). Toxin-associated genes such as *spvB* (29.6%), *pltA* (25.9%), *cdtB* (25.9%), *pltB* (22.2%), and *pltC* (11.1%) were relatively rare (Fig. 2A; Table S2).

### Distribution of ARGs

A total of 1,148 ARGs were identified across the 27 *Salmonella* isolates. The ARGs analysis revealed that resistance genes were associated with multiple antibiotic classes. When classified by antimicrobial class, resistance genes associated with fluoroquinolones were the most abundant (33.0%), followed by aminoglycosides (18.0%), peptide antibiotics (9.3%), and monobactams (9.2%), whereas resistance genes linked to carbapenems, disinfectants, and diaminopyrimidines were relatively rare (Table 1; Fig. S1). Multidrug efflux systems and regulatory components were highly conserved across the isolates. The widespread detection of efflux-associated determinants across all isolates is consistent with their role as intrinsic components of the *Salmonella* core genome, including systems such as *acrAB–TolC* (100%) and global regulators such as *marA* (100%) and *soxR* (100%). In contrast, several acquired resistance determinants showed greater variability among isolates. Aminoglycoside resistance genes displayed heterogeneous distribution, with *aac(6')-Iy* present in 81.5% of isolates, while other aminoglycoside-modifying enzymes, such as *aac(2')-IIa*, *aph(3')-Ia*, and *aadA* variants, occurred at lower frequencies. Tetracycline resistance genes were also detected, including *tet(A)* (33.3%), *tet(C)* (22.2%), and *tet(M)* (7.4%), whereas the multidrug transporter gene *mdfA* was present in all isolates. Additional resistance genes included *sul2* (33.3%), associated with sulfonamide resistance; phenicol resistance genes, such as *floR* (29.6%); and β-lactam resistance genes, including *bla_CMY-2_* (22.2%) and *bla_TEM_* (7.4%) (Fig. 2B; Table S3).

**TABLE 1:** Distribution of antimicrobial resistance mechanisms

Type	Percentage (%)
Antibiotic efflux	62.1
Antibiotic target alteration	13.3
Antibiotic inactivation	10.7
Target alteration + efflux	7.5
Efflux + reduced permeability	2.35
Target replacement	1.74
Target protection	0.4
Fluoroquinolone	33.0
Aminoglycoside	18.0
Peptide	9.3
Monobactam	9.2
Macrolide	7.1
Tetracycline	4.9
Aminocoumarin	4.6
Phosphonic acid	2.9
Nitroimidazole	2.4
Nucleoside	2.4
Phenicol	1.8
Sulfonamide	1.5
Elfamycin	1.3
Carbapenem	0.9
Disinfecting agents	0.4
Diaminopyrimidine	0.3

### Plasmid diversity among *Salmonella* isolates

**Fig 3:**
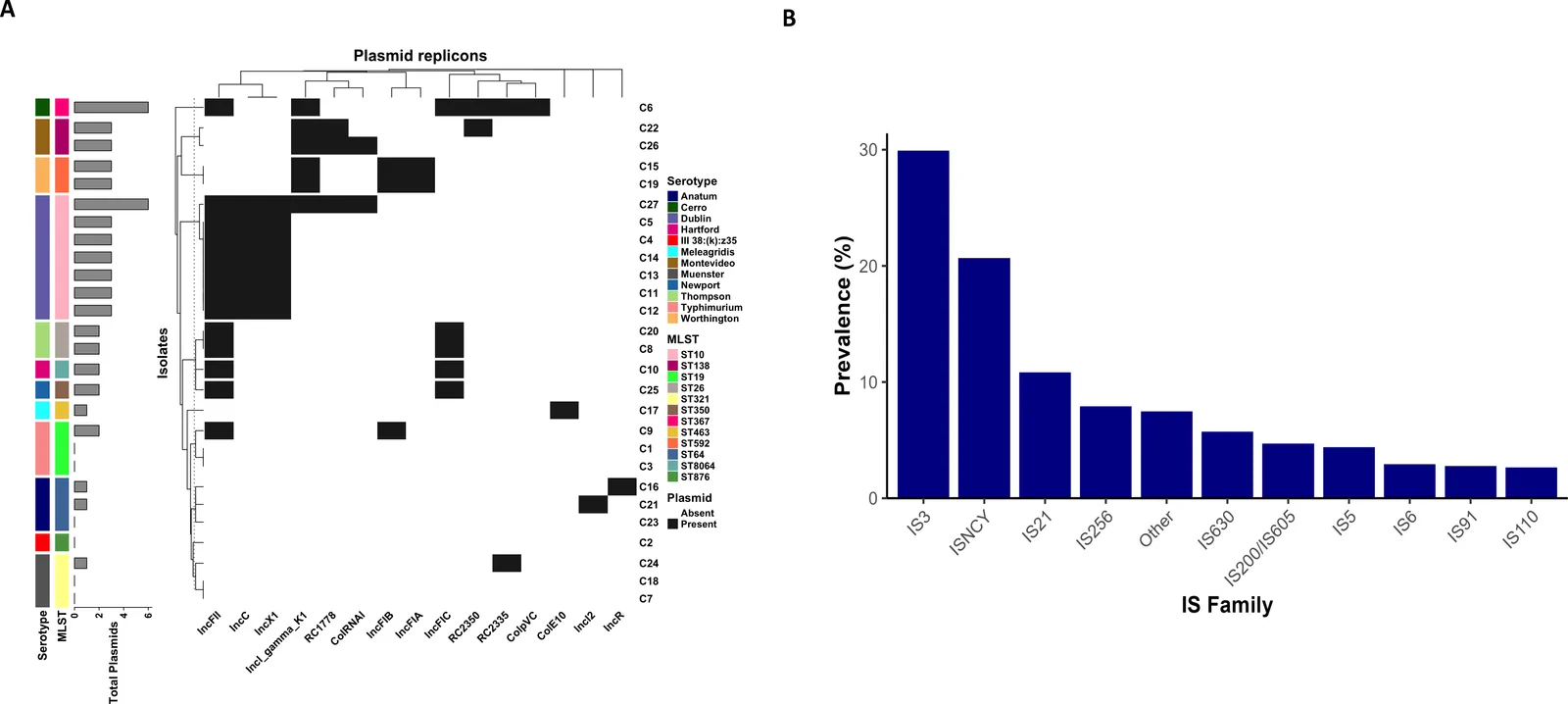
Distribution of plasmid replicons and insertion sequence families among *S. enterica* isolates. (**A**) Heatmap showing the distribution of plasmid replicon families. Rows represent individual isolates, and columns represent plasmid replicon families. Black cells indicate presence, whereas white cells indicate absence. Hierarchical clustering based on replicon profiles is shown above the heatmap. The bar plot on the left shows the total number of replicons detected in each isolate, and the colored annotations indicate serotype and multilocus sequence type (MLST). (**B**) Bar plot showing the prevalence of major insertion sequence (IS) families among the isolates.

Plasmid profiling using MOB-suite identified plasmid replicons in 21 of 27 isolates (77.8%). In total, 15 distinct plasmid replicon types were detected, with several isolates harboring multiple replicons. Among all 27 isolates, the most prevalent replicon type was IncFII, detected in 48.1% (13/27) of the isolates. Other frequently observed incompatibility groups included IncC and IncX1, each present in 25.9% (7/27) of isolates, followed by IncI_gamma_K1 at 22.2% (6/27). Additional plasmid types were identified at lower frequencies, including IncFIC (18.5%; 5/27), IncFIB, and RC1778 (11.1%; 3/27). Several other replicons occurred sporadically, including IncFIA, ColRNAI, RC2335, and RC2350, each detected in 7.4% (2/27) of isolates. Rare plasmid types, including IncI2, IncR, ColE10, and ColpVC, were each identified in a single isolate (3.7%) (Table 2). Several isolates harbored multiple plasmid replicons. The most frequent plasmid replicon configuration comprised IncC, IncFII, and IncX1 replicons, which were detected in several isolates of *S*. Dublin (ST10), including C4, C5, C11–C14, and C27. Additionally, IncF-family plasmids frequently co-occurred, particularly the IncFIC–IncFII combination, which was observed in isolates C8, C10, C20, and C25. The highest plasmid replicon diversity was observed in isolates C6 and C27, each harboring six plasmid replicons (Fig. 3A).

**TABLE 2:** Plasmid replicon distribution among *S. enterica* isolates

Replicon type	No of isolates (*n* = 27)	Percentage (%)
IncFII	13	48.1
IncC	7	25.9
IncX1	7	25.9
IncI_gamma_K1	6	22.2
IncFIC	5	18.5
IncFIB	3	11.1
RC1778	3	11.1
IncFIA	2	7.4
ColRNAI	2	7.4
RC2335	2	7.4
RC2350	2	7.4
IncR	1	3.7
IncI2	1	3.7
ColpVC	1	3.7
ColE10	1	3.7

### Landscape of Mobile genetic elements (MGEs) in *Salmonella* isolates

IntegronFinder analysis identified integron-associated elements in 11.1% (3/27) of *S. enterica* isolates. CALIN structures (clusters of *attC* sites lacking integrase genes) were identified in isolates C6 (*n* = 1 integron), C15 (*n* = 2), and C19 (*n* = 2), whereas complete integrons were detected only in C15 (*n* = 2) and C19 (*n* = 2). In total, five CALIN elements and four complete integrons were identified across the data set (Table 3), indicating a limited distribution of integron-associated elements among isolates. Prophage analysis identified 122 prophage regions across the 27 *S. enterica* isolates. All genomes harbored at least one prophage, with the number of prophage regions per isolate ranging from 1 to 8 (median = 5). Isolates C17 and C24 exhibited the highest prophage burden (*n* = 8), followed by C18 and C7, which each contained seven prophage regions. The number of prophage regions varied across isolates, indicating heterogeneity in prophage content (Fig. S2). We identified 682 IS elements across the 27 *S. enterica* genomes analyzed. The IS burden per isolate ranged from 28 to 106 elements. Eighteen distinct IS families were detected. The IS*3* family was the most prevalent, accounting for 29.9% (*n* = 204) of all detected elements, followed by IS*21* (10.9%, *n* = 74). A substantial proportion of elements (20.7%, *n* = 141) were classified as ISNCY, representing uncharacterized IS elements. Additional families, including IS*256* (7.9%), IS*630* (5.7%), IS*200/IS605* (4.7%), and IS*5* (4.4%), were also identified (Fig. 3B).

**TABLE 3:** Integron-associated elements identified by IntegronFinder in *S. enterica* isolates.

Isolate	CALIN elements	Complete integrons	Integron detected
C6	1	0	Yes
C15	2	2	Yes
C19	2	2	Yes

### Genomic localization of ARGs and their association with MGEs

**Fig 4:**
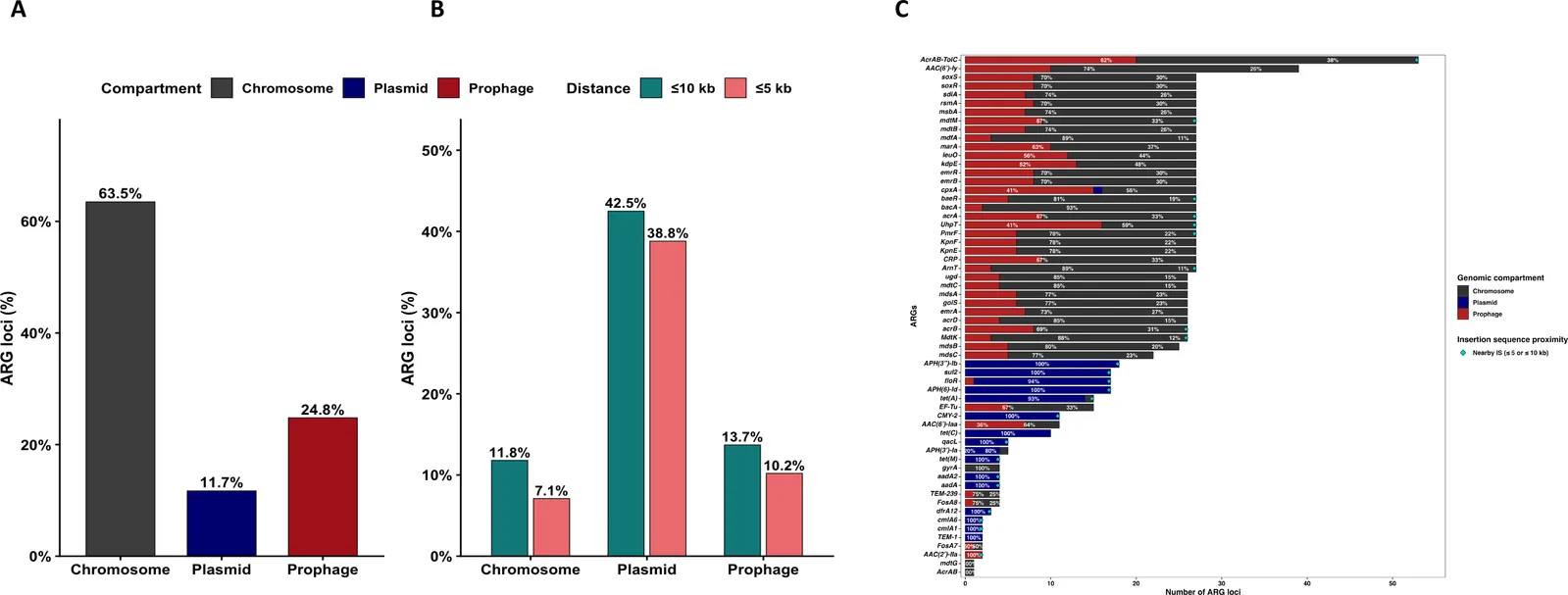
Genomic distribution of antimicrobial resistance genes and their association with insertion sequences. (**A**) Percentage distribution of antimicrobial resistance genes (ARGs) among chromosomal, plasmid, and prophage regions. (**B**) Distribution of ARGs located within 5 kb and 10 kb of insertion sequence (IS) elements. (**C**) Distribution of individual ARGs across genomic compartments and their association with IS elements. Horizontal stacked bars show the number of ARG loci identified in chromosomal, plasmid, and prophage regions. White labels within the bars indicate the percentage contribution of each genomic compartment to the total number of loci detected for each ARG.

The majority of ARG loci were chromosomal (63.5%, *n* = 729/1148), followed by prophage-associated loci (24.8%, *n* = 285/1148) and plasmid-associated loci (11.7%, *n* = 134/1148) (Fig. 4A). IS proximity analysis revealed compartment-specific differences in ARG–IS associations. Plasmid-associated ARGs showed the closest association with IS elements, with 42.5% located within 10 kb and 38.8% within 5 kb. In contrast, chromosomal ARGs exhibited substantially lower proximity (11.8% within 10 kb; 7.1% within 5 kb), while prophage-associated ARGs displayed intermediate levels (13.7% within 10 kb; 10.2% within 5 kb) (Fig. 4B). At the gene level, several ARG classes were frequently located near IS elements. Aminoglycoside resistance genes (*aadA*, *aac(2)*, and *aph* variants) were commonly detected within IS elements. β-lactamase genes, including *bla_TEM_* and *bla_CMY-2_*, also frequently occurred near IS elements, consistent with the role of IS-mediated mobilization in the dissemination of β-lactam resistance. Additional ARGs located near IS elements included tetracycline resistance genes *tet(A)* and *tet(B*) (Fig. 4C; Table S4). ARG–IS family mapping within 10 kb of each other identified gene-specific associations. IS*91* was the predominant IS family associated with genes including *floR*, *sul2*, *tet(A)*, *aph(3″)-Ib* and *aph(6)-Id.*

Conversely, *bla_CMY-2_* was consistently linked to IS*1380*, whereas *aadA2*, *cmlA1*, *cmlA6*, *dfrA12, qacL*, and *tet(M)* were all associated with *IS256* (Fig. S3). Screening for SGI1/SGI2 extracted 285 chromosomal ARG-containing contigs, representing 729 chromosomal ARG entries. BLASTN analysis detected only short partial matches to SGI reference sequences. Because these matches exhibited very low query coverage, ranging from approximately 0.10% to 3.98%, they were not considered convincing evidence of SGI-like resistance islands. Consequently, no chromosomal ARG-containing contig met the criteria for classification as SGI-like.

### Pan-genome structure

**Fig 5:**
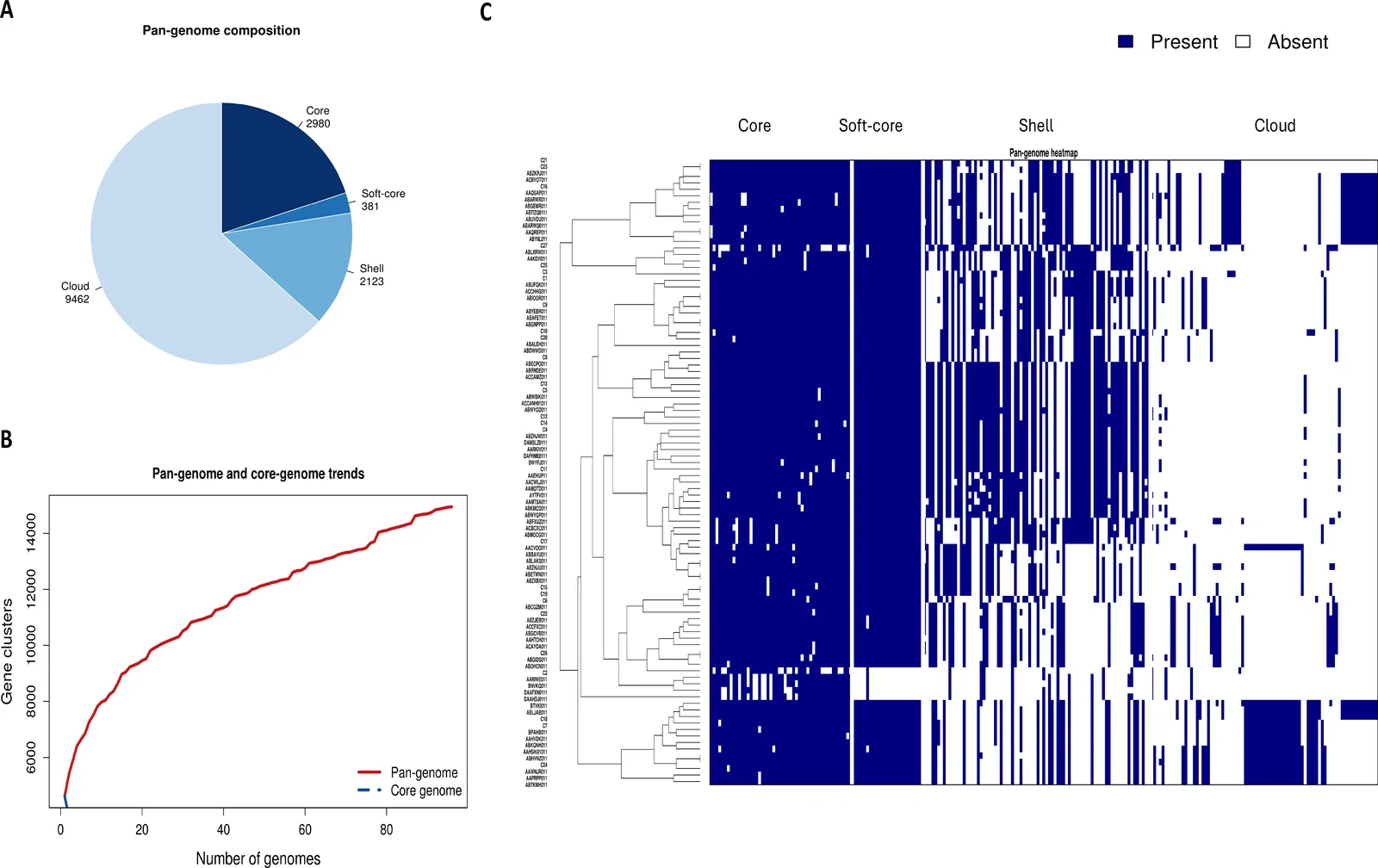
Pan-genome structure and gene-cluster distribution among 124 *S. enterica* genomes. (**A**) Distribution of 14,946 gene clusters across pan-genome categories: 2,980 core genes (19.9%), 381 soft-core genes (2.5%), 2,123 shell genes (14.2%), and 9,462 cloud genes (63.3%). (**B**) Pan-genome and core-genome accumulation curves generated using the 27 study isolates and 97 publicly available *S. enterica* genomes retrieved from NCBI. (**C**) Heatmap showing the distribution of gene clusters across the 124 genomes. Rows represent individual genomes hierarchically clustered based on gene-content similarity, whereas columns represent gene clusters identified in the pan-genome analysis.

Pan-genome analysis of 124 *S. enterica* isolates identified 14,946 gene clusters. Among these, 2,980 genes (19.9%) constituted the core genome, indicating a conserved set of functions across the isolates, while an additional 381 genes (2.5%) were classified as soft-core genes. The accessory genome represented most of the gene repertoire (11,585 genes; 77.6%), including 2,123 shell genes (14.2% of the pan-genome) and 9,462 cloud genes (63.3% of the pan-genome) (Fig. 5A). Gene accumulation analysis revealed a steady increase in pan-genome size with each additional genome, whereas the core genome plateaued after incorporating multiple isolates (Fig. 5B). The distribution of gene clusters across genomes is further illustrated by the presence–absence heatmap, highlighting conserved core regions and extensive variability within the accessory genome (Fig. 5C).

### Core genome-based phylogeny

**Fig 6:**
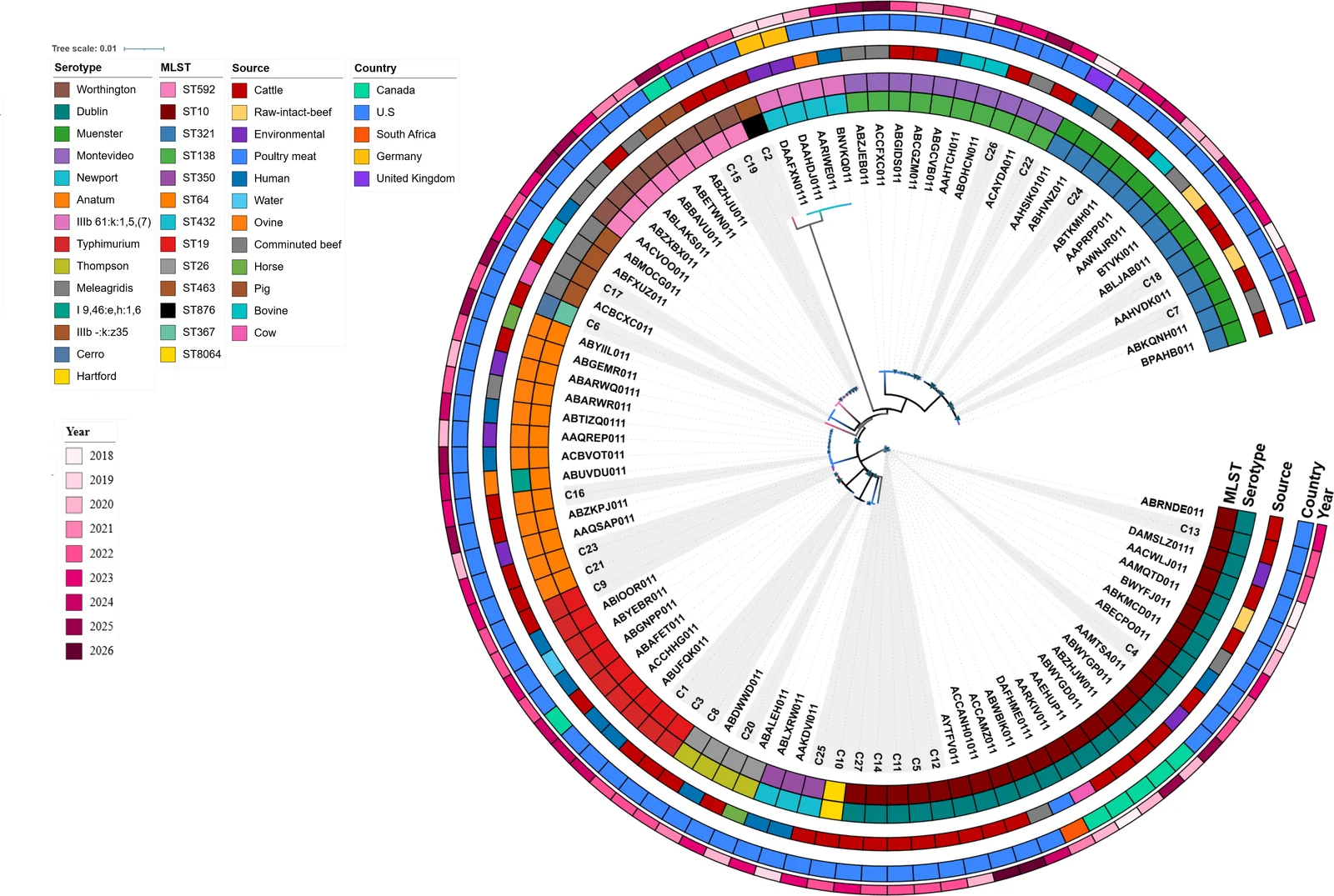
Phylogenetic relationships among 124 *S. enterica* genomes, including 27 study isolates and 97 publicly available genomes retrieved from NCBI and representing diverse sources. From the innermost to the outermost ring, the concentric annotation tracks indicate isolate identity, serotype, multilocus sequence type (MLST), source, country of origin, and year of isolation. The phylogenetic tree was constructed using the neighbor-joining method based on Jukes–Cantor nucleotide distances, with 1,000 bootstrap replicates.

Core genome phylogenetic analysis of 124 *S. enterica* genomes provided a high-resolution view of the evolutionary relationships among the analyzed isolates. Notably, large clusters were observed for serotypes such as *S*. Worthington (ST592), *S*. Dublin (ST10), and *S*. Typhimurium (ST19). The ST10 (*S*. Dublin) isolates formed a tightly clustered, well-supported group characterized by short branch lengths and high bootstrap support. In contrast, isolates from other sequence types, including ST19 (*S*. Typhimurium), ST64 (*S*. Anatum), and ST321 (*S*. Muenster), were more phylogenetically dispersed and frequently interspersed with isolates from diverse sources. Importantly, our isolates clustered alongside genomes from diverse sources, including food-, environment-, and human-associated data sets. Such clustering suggests cross-sector dissemination (Fig. 6).

## DISCUSSION

The rapid increase in AMR has significantly reshaped the epidemiology of many foodborne pathogens (18). Among these, *S. enterica* remains a major concern due to its ability to acquire and disseminate multiple ARGs across different hosts and environmental reservoirs (18, 48). Accordingly, genomic characterization of cattle-associated *Salmonella* provides important insights into ARG profiles, virulence factors, and MGEs content. In this study, we performed a comprehensive genomic characterization of *Salmonella* isolates that had previously been phenotypically assessed for AMR and virulence-associated traits (23). MLST analysis showed associations between several serotypes and ST lineages, including *S*. Dublin (ST10), *S*. Typhimurium (ST19), *S*. Anatum (ST64), and *S*. Muenster (ST321) (Fig. 3; Table S1). Notably, 25.9% of the isolates belonged to ST10, a globally recognized, cattle-adapted lineage of *S*. Dublin (18).

Furthermore, WGS enabled high-resolution functional characterization, identifying virulence-associated genes across isolates (Fig. 2A; Table S2). The universal presence of biofilm-associated genes (100%), particularly those of the *csg* operon (*csgA*, *csgB*, *csgD*), was consistent with our phenotypic findings, showing that all isolates exhibited biofilm-forming ability (23). This concordance highlights the potential role of curli-mediated biofilm formation in supporting environmental persistence and host colonization (49). Similarly, key components of the T3SS encoded within SPIs, including *invA*, *sipB*, and *sopB*, were broadly conserved across all isolates, reflecting their essential roles in host-cell invasion and intracellular survival (23, 50). In contrast, iron acquisition genes showed greater variability among isolates, indicating differences in the genetic repertoire underlying iron-scavenging mechanisms. Toxin-associated genes were also detected at relatively low frequencies, suggesting that classical toxin production may not be the predominant virulence strategy in these isolates (18). Overall, variation in accessory virulence genes may influence bacterial fitness under different host or environmental conditions and may contribute to differences in virulence potential and disease outcomes, as suggested by previous studies (51, 52).

WGS identified genetic determinants that may contribute to the MDR phenotypes observed in our previous antimicrobial susceptibility testing (23, 53). The observed phenotypic MDR profiles align with the presence of conserved multidrug efflux systems, which serve as a primary mechanism of intrinsic resistance. These systems actively extrude diverse antimicrobial compounds, including fluoroquinolones and β-lactams, thereby reducing intracellular drug concentrations (54–56). The emrAB system (*emrA*: 96.3% and *emrB:* 100%), previously associated with resistance to macrolides and phenicols, may further contribute to the widespread resistance to azithromycin and clindamycin, which was observed in 100% of isolates. Additionally, the resistance to tetracycline and doxycycline observed in the susceptibility assays is consistent with the presence of the *tet(A), tet(C)*, and *tet(M)* genes (Table S3, Fig. S1). Likewise, the detection of *floR* corresponds with the chloramphenicol resistance observed in many isolates. Aminoglycoside resistance was associated with genes such as *aac_(6)-Iy_* and other aminoglycoside-modifying enzymes, whereas *aac_(6)-Iaa_*, although detected, is considered a cryptic gene with limited phenotypic relevance (57, 58). The 100% phenotypic resistance to neomycin and gentamicin reported previously was consistent with the presence of the aminoglycoside-associated efflux pump gene *acrD* (96.3%) and its core regulatory genes, including *baeR* (100%), *cpxA* (100%), and *kdpE* (100%), together with acquired phosphotransferases such as *aph(3*′′*)-Ib* (33.3%) in specific MDR lineages (23, 59, 60).

Plasmids represent major drivers of horizontal gene transfer and play a critical role in the dissemination of AMR in *S. enterica* (18)*.* Plasmid replicons were detected in 21 of 27 isolates (77.8%), with 15 distinct types identified (Table 2). The identification of multiple incompatibility groups highlights the diversity of plasmid backbones. IncF-family plasmids, particularly IncFII, were the most prevalent (48.1%). Consistent with McMillan et al. (2019), IncF-type replicons are prevalent in *Salmonella* from U.S. food animals and support stable carriage of resistance genes (61). The most frequent plasmid configuration comprised IncC, IncFII, and IncX1 replicons and was detected in several isolates of the *S.* Dublin (ST10) lineage (Fig. 3A). Similar plasmid architectures have been reported in previous genomic studies of *S.* Dublin isolates, which are commonly associated with IncX1 and IncFII replicons and MDR plasmids such as IncC, all of which contribute to the persistence and pathogenic potential of this lineage (62–64). Comparison of ST10/*S.* Dublin and ST19/*S.* Typhimurium showed broadly overlapping ARG classes and conservation of most virulence categories. Although ST10/*S.* Dublin represents a cattle-adapted lineage, it did not exhibit reduced ARG or virulence-class diversity in this cohort. Plasmid-associated virulence genes were consistently detected in ST10 but varied among ST19 isolates, suggesting that lineage-specific differences may reflect differences in individual gene content, plasmid association, or accessory genome organization rather than in broad functional classes. However, the limited number of isolates per lineage restricts broader conclusions regarding the relationship between host adaptation and accessory-genome diversity.

IntegronFinder analysis further revealed the presence of integron-associated elements in 11.1% of the isolates. CALIN structures were identified in C6, C15, and C19, whereas complete integrons were confined to C15 and C19, indicating that these isolates possessed site-specific recombination systems with the potential to capture and express resistance gene cassettes (Table 3) (65). To further explore the genomic context of these isolates, we examined prophage regions and IS elements. The identification of 122 prophage regions and 682 IS elements across the 27 *Salmonella* isolates highlights a genome shaped by horizontal gene transfer and internal remodeling. Notably, isolate C6 exhibited a complex mobilome, with similar patterns observed in C15 and C19, while C27 showed a notably high IS burden alongside multiple plasmids (Fig. S2). Isolates with high MGE burdens may serve as reservoirs of genomic variation and adaptive traits under antimicrobial selective pressure (66, 67).

In this study, most ARG loci were located on the chromosome (63.5%), whereas smaller proportions were associated with prophage regions (24.8%) or plasmids (11.7%). Despite the lower overall proportion of plasmid-associated ARGs, plasmids are notably associated with IS elements. Nearly half of plasmid-borne ARG loci (42.5%) were located within 10 kb of an IS element, and 38.8% were located within 5 kb (Fig. 4A and B). The close proximity of plasmid-associated ARGs to IS elements suggests increased potential for IS-mediated mobilization and genomic rearrangement within plasmid backbones (68, 69). In addition to mobilizing resistance determinants, IS elements may influence the expression of adjacent genes by introducing promoters or forming hybrid promoters with neighboring host sequences (70, 71). Gene-level analysis in this study showed that β-lactamase genes, particularly *bla*_*TEM*_ and *bla_CMY-2_ were* frequently located in proximity to IS on plasmids. Notably, *bla*_*TEM*_ is commonly associated with Tn3-family transposons and often occurs adjacent to IS elements, a genomic configuration known to facilitate both mobilization and expression of resistance determinants (72, 73). This pattern is consistent with earlier studies reporting that plasmids facilitate the mobilization of resistance genes, whereas chromosomal integration contributes to their long-term stability (47, 74). IS-mediated transposition may provide a mechanism by which resistance determinants move between plasmid and chromosomal contexts; however, such movement was not directly demonstrated in this study (75, 76).

In this study, IS*91* was the predominant IS family associated with acquired ARGs, including *floR*, *sul2*, *tet (A)*, *aph(3*′′*)-Ib*, and *aph (6)-Id*. This pattern is consistent with reports of ESBL-producing *E. coli*, in which the same genes, along with *bla_TEM-1A_*, were co-located on IS*91*-associated contigs (77). Similarly, cattle-associated *S. enterica* studies described the *floR–tet(A)–aph (6)-Id–aph(3*′′*)-Ib–sul2* cluster flanked by IS*Vsa3*, an IS*91*-like transposase (78). This association is biologically plausible because IS*91* is frequently located near ARGs and has been implicated in their mobilization. Through rolling-circle transposition, incomplete termination can co-mobilize adjacent DNA (79). The consistent association of *bla_CMY-2_* with an IS*1380*-family element in our dataset agrees with previous reports linking *bla_CMY-2_* to ISEcp1, a member of the IS*1380* family (80). In MDR *Salmonella* of swine origin, *bla_CMY-2_* was located adjacent to ISEcp1, and the conserved ISEcp1–*bla_CMY-2_* structure has been reported across different plasmid backbones. Thus, our finding that all IS-proximal *bla_CMY-2_* occurrences were associated with IS*1380* supports the role of ISEcp1/IS*1380* as an important mobile context for AmpC β-lactamase dissemination (81). The clustering of IS*256* with *aadA2*, *cmlA1*, *dfrA12*, *tet (A)*, and *qacL* suggests an integron-associated MDR region rather than isolated ARG acquisition. Similar structures have been reported in MDR *S. enterica* from companion animals, where class 1 integrons and AMR regions included *qacL–IS256–sul3* arrangements and related resistance genes (82). In addition, IS elements can influence nearby resistance genes by providing promoter sequences or forming hybrid promoters; IS*256* has specifically been described among IS families capable of hybrid promoter formation (75, 83).

Consistently, our pan-genome analysis revealed that the *S. enterica* dataset is dominated by accessory genes (77.6%), whereas the core genome accounts for 19.9%. A large fraction of the pan-genome consisted of cloud genes (63.3%), indicating extensive variability in gene content among isolates (Fig. 5A and B). Such gene distribution patterns are consistent with previous comparative genomic studies showing that *Salmonella* populations possess open pan-genomes shaped by frequent gene gain and loss events (84, 85). The substantial cloud gene fraction likely reflects ongoing acquisition of genetic material through horizontal gene transfer and AMR evolution, as well as adaptation to diverse ecological niches (18). The phylogenetic analysis in this study indicated that AMR in this population was distributed among multiple lineages rather than being restricted to the clonal expansion of a single lineage. Core genome phylogeny revealed distinct lineage clustering alongside broad ecological relatedness. Notably, ST10 (*S.* Dublin) isolates form a related cluster with low genetic diversity (Fig. 6). This pattern is consistent with the persistence of a relatively stable, cattle-adapted lineage but does not independently demonstrate long-term transmission or persistence (18). The Kentucky bovine isolates also clustered with North American clinical, food, and environmental strains. These findings indicate that closely related lineages occur across animal, food, environmental, and human-associated sources. Although the data do not establish direct transmission events, they support broader epidemiological connectivity across One Health compartments (86).

These findings suggest that the cattle-associated *Salmonella* may form part of a wider network of MDR lineages occurring across interconnected sources, emphasizing the importance of integrated surveillance. The chromosome may serve as a relatively stable reservoir because it harbored 63.5% of resistance loci, whereas the strong association of plasmid-associated ARGs with IS elements highlights a dynamic mobilome with the potential for genomic adaptation and resistance dissemination. These findings reinforce the need for strategies that reduce antimicrobial selection pressure and limit the persistence and spread of MDR lineages (87, 88). Although WGS defines the AMR profile, it does not confirm phenotypic expression or active transposition within the stressful environment of a bovine host. Consequently, future research may involve functional and transcriptomic analyses to elucidate how these elements are regulated during active infection (89, 90).

In summary, our findings indicate that MDR *S. enterica* was present among necropsied cattle in this cohort, highlighting potential One Health significance and the need for continued surveillance. The MDR phenotypes observed in this cohort, together with the conservation of the ST10 (*S.* Dublin) lineage, supports the clinical and diagnostic challenges posed by persistent, host-adapted populations in livestock. The close phylogenetic relatedness between these cattle isolates and contemporary clinical and environmental strains supports the persistence of shared lineages across interconnected animal, food, and human-associated systems. The genomic architecture of these isolates reflects a dual framework: a stable chromosomal reservoir of resistance genes coupled with a dynamic mobilome shaped by plasmids, prophages, and IS elements. This configuration supports both the long-term maintenance of resistance and the potential for horizontal dissemination of ARGs and virulence genes. Given the high mortality associated with *S.* Dublin in cattle and the recognized severity of human infections, the emergence of these MDR lineages represents a critical intersection of veterinary and public health concerns. Addressing this threat requires a shift from passive monitoring to proactive One Health surveillance and mobilome tracking to disrupt genetic transmission at the animal–food interface. A more comprehensive epidemiological assessment will require the inclusion of healthy cattle and systematic, population-based sampling across the Kentucky bovine population. Accordingly, the 27 isolates analyzed here should be interpreted as a diagnostic clinical subset from central Kentucky rather than a statewide estimate of the bovine *Salmonella* reservoir. This study has several limitations. The isolates were obtained from necropsied cattle submitted to a diagnostic laboratory and therefore may not represent the broader cattle population in Kentucky. In addition, short-read sequencing may not fully resolve plasmids, prophages, repetitive regions, or ARG–MGE linkages. Finally, genomic detection alone does not confirm gene expression, active transposition, phenotypic effects, or direct transmission. Future studies incorporating long-read or hybrid sequencing, together with functional genomic and metagenomic approaches, will improve the resolution of resistance architecture and help identify actively expressed resistance mechanisms and the MGEs that contribute to their dissemination. Coordinated surveillance, antimicrobial stewardship, and prevention strategies across veterinary and public health sectors remain essential for preserving the effectiveness of clinically important antimicrobials.

## Data Availability

All raw sequences data were submitted to the NCBI Sequence Read Archive under the BioProjects PRJNA1086499 and PRJNA1237852 (https://www.ncbi.nlm.nih.gov/sra). All supporting data have been provided within this article or in the supplemental material files.

## SUPPLEMENTAL MATERIAL

File S1 spectrum.01581-26-s0001.xlsx File S1.

Supplemental figures spectrum.01581-26-s0002.pptx Fig. S1 to S3.

Supplemental tables spectrum.01581-26-s0003.docx Tables S1 to S4.

ASM does not own the copyrights to Supplemental Material that may be linked to, or accessed through, an article. The authors have granted ASM a non-exclusive, world-wide license to publish the Supplemental Material files. Please contact the corresponding author directly for reuse.
