# Supplementary material for: Genomic architecture of antimicrobial resistance and mobile genetic elements in Salmonella enterica isolated from necropsied cattle in central Kentucky
Source: Microbiol Spectr. 2026 Aug 31;14(10):e01581-26. doi: 10.1128/spectrum.01581-26 (PMC13689396; doi:10.1128/spectrum.01581-26)
Supplement: Supplementary file 1 [file spectrum.01581-26-s0003.docx]

**Table S1**. Genome Assembly Statistics, Serotype, and MLST Profiles of S. enterica Isolates

| Isolate | Serotype | MLST | Genome Size (Mb) | Contigs | GC (%) |
| --- | --- | --- | --- | --- | --- |
| C1 | Typhimurium | ST19 | 4.87 | 90 | 52.1 |
| C2 | III 38:(k): z35 | ST876 | 5.06 | 73 | 51.4 |
| C3 | Typhimurium | ST19 | 4.82 | 47 | 52.2 |
| C4 | Dublin | ST10 | 4.97 | 45 | 52.1 |
| C5 | Dublin | ST10 | 5.02 | 42 | 52.1 |
| C6 | Cerro | ST367 | 4.71 | 69 | 52.2 |
| C7 | Muenster | ST321 | 4.71 | 33 | 52.1 |
| C8 | Thompson | ST26 | 4.74 | 44 | 52.1 |
| C9 | Typhimurium | ST19 | 4.88 | 51 | 52.2 |
| C10 | Hartford | ST8064 | 4.75 | 74 | 52.1 |
| C11 | Dublin | ST10 | 4.98 | 67 | 52.1 |
| C12 | Dublin | ST10 | 5.01 | 47 | 52.1 |
| C13 | Dublin | ST10 | 5.07 | 50 | 52.1 |
| C14 | Dublin | ST10 | 4.99 | 74 | 52.1 |
| C15 | Worthington | ST592 | 5.12 | 58 | 52.2 |
| C16 | Anatum | ST64 | 4.80 | 36 | 52.2 |
| C17 | Meleagridis | ST463 | 4.94 | 64 | 52.0 |
| C18 | Muenster | ST321 | 4.78 | 33 | 52.2 |
| C19 | Worthington | ST592 | 5.09 | 61 | 52.2 |
| C20 | Thompson | ST26 | 4.78 | 32 | 52.1 |
| C21 | Anatum | ST64 | 4.78 | 26 | 52.0 |
| C22 | Montevideo | ST138 | 4.61 | 37 | 52.2 |
| C23 | Anatum | ST64 | 4.67 | 32 | 52.1 |
| C24 | Muenster | ST321 | 4.86 | 27 | 52.2 |
| C25 | Newport | ST350 | 4.86 | 40 | 52.1 |
| C26 | Montevideo | ST138 | 4.68 | 42 | 52.1 |
| C27 | Dublin | ST10 | 5.4 | 370 | 52.1 |

**Table S2.** Distribution and prevalence of virulence-associated genes identified in S. enterica isolates.

| **Virulence class** | **Gene** | **No. of isolates (n)** | **Prevalence (%)** |
| --- | --- | --- | --- |
| **Adhesion/fimbriae** | *fimA* | 27 | 100 |
|  | *fimD* | 27 | 100 |
|  | *fimH* | 27 | 100 |
|  | *fimW* | 27 | 100 |
|  | *fimZ* | 27 | 100 |
|  | *fimC* | 26 | 96.3 |
|  | *fimF* | 26 | 96.3 |
|  | *fimI* | 26 | 96.3 |
|  | *fimY* | 26 | 96.3 |
|  | *pagN* | 26 | 96.3 |
|  | *sinH* | 26 | 96.3 |
|  | *misL* | 25 | 92.6 |
|  | *lpfA* | 17 | 63 |
|  | *lpfC* | 17 | 63 |
|  | *lpfE* | 17 | 63 |
|  | *lpfB* | 14 | 51.9 |
|  | *lpfD* | 14 | 51.9 |
|  | *shdA* | 4 | 14.8 |
|  | *pefA* | 1 | 3.7 |
|  | *pefB* | 1 | 3.7 |
|  | *pefC* | 1 | 3.7 |
|  | *pefD* | 1 | 3.7 |
|  | *rck* | 1 | 3.7 |
| **Biofilm / matrix** | *csgA* | 27 | 100 |
|  | *csgB* | 27 | 100 |
|  | *csgC* | 27 | 100 |
|  | *csgD* | 27 | 100 |
|  | *csgE* | 27 | 100 |
|  | *csgG* | 27 | 100 |
|  | *csgF* | 26 | 96.3 |
|  | *bapA* | 20 | 74.1 |
| **Intracellular survival** | *steC* | 27 | 100 |
|  | *steD* | 27 | 100 |
|  | *sifB* | 26 | 96.3 |
|  | *slrP* | 26 | 96.3 |
|  | *steA* | 26 | 96.3 |
|  | *steB* | 26 | 96.3 |
|  | *pipB* | 25 | 92.6 |
|  | *sifA* | 25 | 92.6 |
|  | *pipB2* | 21 | 77.8 |
|  | *gtgE* | 12 | 44.4 |
|  | *srfJ* | 11 | 40.7 |
|  | *sspH2* | 9 | 33.3 |
|  | *gogB* | 5 | 18.5 |
|  | *sspH1* | 2 | 7.4 |
| **Iron acquisition** | *entB* | 25 | 92.6 |
|  | *fepC* | 24 | 88.9 |
|  | *fepG* | 24 | 88.9 |
|  | *entA* | 22 | 81.5 |
|  | *entE* | 2 | 7.4 |
|  | *iucA* | 1 | 3.7 |
|  | *iucB* | 1 | 3.7 |
|  | *iucC* | 1 | 3.7 |
|  | *iucD* | 1 | 3.7 |
|  | *irp1* | 1 | 3.7 |
|  | *irp2* | 1 | 3.7 |
|  | *iutA* | 1 | 3.7 |
|  | *ybtA* | 1 | 3.7 |
|  | *ybtE* | 1 | 3.7 |
|  | *ybtP* | 1 | 3.7 |
|  | *ybtQ* | 1 | 3.7 |
|  | *ybtS* | 1 | 3.7 |
|  | *ybtT* | 1 | 3.7 |
|  | *ybtU* | 1 | 3.7 |
|  | *ybtX* | 1 | 3.7 |
| **Motility/chemotaxis** | *cheR* | 27 | 100 |
|  | *cheY* | 27 | 100 |
|  | *cheZ* | 27 | 100 |
|  | *flgC* | 27 | 100 |
|  | *flgG* | 27 | 100 |
|  | *flgH* | 27 | 100 |
|  | *fliI* | 27 | 100 |
|  | *fliN* | 27 | 100 |
|  | *motA* | 27 | 100 |
|  | *cheB* | 26 | 96.3 |
|  | *cheW* | 26 | 96.3 |
|  | *flhA* | 26 | 96.3 |
|  | *flhC* | 26 | 96.3 |
|  | *fliG* | 26 | 96.3 |
|  | *fliM* | 26 | 96.3 |
|  | *fliQ* | 23 | 85.2 |
|  | *flgI* | 18 | 66.7 |
|  | *flgB* | 7 | 25.9 |
|  | *flhD* | 1 | 3.7 |
| **Regulation** | *fur* | 27 | 100 |
|  | *pmrA* | 27 | 100 |
|  | *rcsB* | 27 | 100 |
|  | *hilA* | 27 | 100 |
|  | *hilD* | 27 | 100 |
|  | *phoP* | 27 | 100 |
|  | *phoQ* | 27 | 100 |
|  | *pmrB* | 26 | 96.3 |
|  | *hilC* | 26 | 96.3 |
| **Secretion systems** | *tssJ* | 26 | 96.3 |
|  | *tssK* | 23 | 85.2 |
|  | *vgrG* | 21 | 77.8 |
|  | *tae4* | 20 | 74.1 |
|  | *tlde1* | 20 | 74.1 |
|  | *hcp2/tssD2* | 7 | 25.9 |
|  | *tssL* | 7 | 25.9 |
|  | *tssM* | 7 | 25.9 |
| **Stress / immune evasion** | *mgtC* | 27 | 100 |
|  | *mig-14* | 27 | 100 |
|  | *sodCI* | 10 | 37 |
|  | *mig-5* | 7 | 25.9 |
| **T3SS / SPI** | *sscA* | 27 | 100 |
|  | *sscB* | 27 | 100 |
|  | *sseA* | 27 | 100 |
|  | *sseB* | 27 | 100 |
|  | *sseD* | 27 | 100 |
|  | *sseE* | 27 | 100 |
|  | *sseJ* | 27 | 100 |
|  | *ssrB* | 27 | 100 |
|  | *invA* | 27 | 100 |
|  | *invB* | 27 | 100 |
|  | *invC/sctN* | 27 | 100 |
|  | *invF* | 27 | 100 |
|  | *invH* | 27 | 100 |
|  | *invI* | 27 | 100 |
|  | *orgA/sctK* | 27 | 100 |
|  | *orgB/SctL* | 27 | 100 |
|  | *orgC* | 27 | 100 |
|  | *prgH* | 27 | 100 |
|  | *prgI* | 27 | 100 |
|  | *prgK* | 27 | 100 |
|  | *sipA/sspA* | 27 | 100 |
|  | *sipB/sspB* | 27 | 100 |
|  | *sipC/sspC* | 27 | 100 |
|  | *sopE2* | 27 | 100 |
|  | *spaO/sctQ* | 27 | 100 |
|  | *spaP* | 27 | 100 |
|  | *spaQ* | 27 | 100 |
|  | *spaR* | 27 | 100 |
|  | *spaS* | 27 | 100 |
|  | *spiC/ssaB* | 27 | 100 |
|  | *ssaC* | 27 | 100 |
|  | *ssaD* | 27 | 100 |
|  | *ssaG* | 27 | 100 |
|  | *ssaH* | 27 | 100 |
|  | *ssaK* | 27 | 100 |
|  | *ssaL* | 27 | 100 |
|  | *ssaM* | 27 | 100 |
|  | *ssaP* | 27 | 100 |
|  | *ssaR* | 27 | 100 |
|  | *ssaS* | 27 | 100 |
|  | *ssaX* | 27 | 100 |
|  | *sseC* | 26 | 96.3 |
|  | *sseF* | 26 | 96.3 |
|  | *sseG* | 26 | 96.3 |
|  | *invE* | 26 | 96.3 |
|  | *invG* | 26 | 96.3 |
|  | *invJ* | 26 | 96.3 |
|  | *prgJ* | 26 | 96.3 |
|  | *sipD* | 26 | 96.3 |
|  | *sopB/sigD* | 26 | 96.3 |
|  | *sopD* | 26 | 96.3 |
|  | *ssaE* | 26 | 96.3 |
|  | *ssaI* | 26 | 96.3 |
|  | *ssaJ* | 26 | 96.3 |
|  | *ssaN* | 26 | 96.3 |
|  | *ssaO* | 26 | 96.3 |
|  | *ssaQ* | 26 | 96.3 |
|  | *ssaT* | 26 | 96.3 |
|  | *ssaU* | 26 | 96.3 |
|  | *ssaV* | 26 | 96.3 |
|  | *sseL* | 25 | 92.6 |
|  | *ssrA* | 25 | 92.6 |
|  | *sopD2* | 25 | 92.6 |
|  | *sopA* | 24 | 88.9 |
|  | *sseK1* | 20 | 74.1 |
|  | *sseK2* | 12 | 44.4 |
|  | *sseI/srfH* | 11 | 40.7 |
| **Toxins** | *spvB* | 8 | 29.6 |
|  | *pltA* | 7 | 25.9 |
|  | *cdtB* | 7 | 25.9 |
|  | *pltB* | 6 | 22.2 |
|  | *pltC* | 3 | 11.1 |
| **Others** | *acrB* | 27 | 100 |
|  | *iacP* | 27 | 100 |
|  | *mgtB* | 27 | 100 |
|  | *sicA* | 27 | 100 |
|  | *sicP* | 27 | 100 |
|  | *sprB* | 27 | 100 |
|  | *ompA* | 27 | 100 |
|  | *STM0269* | 26 | 96.3 |
|  | *STM0270* | 26 | 96.3 |
|  | *STM0271* | 26 | 96.3 |
|  | *espO1-1* | 26 | 96.3 |
|  | *galF* | 26 | 96.3 |
|  | *gndA* | 26 | 96.3 |
|  | *iagB* | 26 | 96.3 |
|  | *ibeC* | 26 | 96.3 |
|  | *sptP* | 26 | 96.3 |
|  | *STM0266* | 25 | 92.6 |
|  | *STM0267* | 25 | 92.6 |
|  | *STM0268* | 25 | 92.6 |
|  | *STM0272* | 25 | 92.6 |
|  | *STM0273* | 25 | 92.6 |
|  | *STM0274* | 25 | 92.6 |
|  | *rpoS* | 25 | 92.6 |
|  | *STM0276* | 24 | 88.9 |
|  | *STM0275* | 23 | 85.2 |
|  | *STM0283* | 23 | 85.2 |
|  | *STM0285* | 23 | 85.2 |
|  | *STM0282* | 22 | 81.5 |
|  | *STM0286* | 22 | 81.5 |
|  | *avrA* | 22 | 81.5 |
|  | *STM0284* | 21 | 77.8 |
|  | *STM0290* | 21 | 77.8 |
|  | *siiE* | 21 | 77.8 |
|  | *tre* | 21 | 77.8 |
|  | *STM0278* | 20 | 74.1 |
|  | *STM0287* | 20 | 74.1 |
|  | *allB* | 20 | 74.1 |
|  | *ratB* | 18 | 66.7 |
|  | *STM0279* | 16 | 59.3 |
|  | *spvC* | 8 | 29.6 |
|  | *spvD* | 8 | 29.6 |
|  | *tcpC* | 3 | 11.1 |
|  | *artA* | 2 | 7.4 |
|  | *artB* | 2 | 7.4 |
|  | *fyuA/psn* | 1 | 3.7 |

**Table S3.** Distribution and prevalence of ARGs identified in S. enterica isolates.

| **Resistance category** | **Gene** | **No. of isolates (n=27)** | **Prevalence (%)** |
| --- | --- | --- | --- |
| **Multidrug efflux pump** | *mdtB* | 27 | 100 |
|  | *mdtC* | 26 | 96.3 |
|  | *acrD* | 26 | 96.3 |
|  | *mdtK* | 26 | 96.3 |
|  | *acrA* | 27 | 100 |
|  | *acrB* | 26 | 96.3 |
|  | *emrA* | 26 | 96.3 |
|  | *emrB* | 27 | 100 |
|  | *mdtM* | 27 | 100 |
|  | *KpnE* | 27 | 100 |
|  | *KpnF* | 27 | 100 |
|  | *mdsA* | 26 | 96.3 |
|  | *mdsB* | 25 | 92.5 |
|  | *mdsC* | 22 | 81.5 |
|  | *msbA* | 27 | 100 |
|  | *mdtG* | 1 | 3.7 |
|  | *mdfA* | 27 | 100 |
|  | *AcrAB-TolC* | 27 | 100 |
| **Efflux regulation** | *baeR* | 27 | 100 |
|  | *cpxA* | 27 | 100 |
|  | *kdpE* | 27 | 100 |
|  | *emrR* | 27 | 100 |
|  | *marA* | 27 | 100 |
|  | *rsmA* | 27 | 100 |
|  | *sdiA* | 27 | 100 |
|  | *soxR* | 27 | 100 |
|  | *soxS* | 27 | 100 |
|  | *CRP* | 27 | 100 |
|  | *golS* | 26 | 96.3 |
|  | *leuO* | 27 | 100 |
| **Aminoglycoside** | *aac(2')-IIa* | 1 | 3.7 |
|  | *aac(6')-Iaa* | 5 | 18.5 |
|  | *aac(6')-Iy* | 22 | 81.5 |
|  | *aph(3'')-Ib* | 9 | 33.3 |
|  | *aph(3')-Ia* | 3 | 11.1 |
|  | *aph(6)-Id* | 9 | 33.3 |
|  | *aadA* | 2 | 7.41 |
|  | *aadA2* | 2 | 7.4 |
| **β-lactam** | *blaCMY-2* | 6 | 22.2 |
|  | *blaTEM-1* | 1 | 3.7 |
|  | *blaTEM-239* | 4 | 14.8 |
| **Diaminopyrimidine** | *dfrA12* | 2 | 7.4 |
| **Disinfectants and antiseptics** | *qacL* | 2 | 7.4 |
| **Elfamycin** | *EF-Tu* | 10 | 37.0 |
| **Fluoroquinolone** | *gyrA* | 4 | 14.8 |
| **Peptide** | *arnT* | 27 | 100 |
|  | *PmrF* | 27 | 100 |
|  | *bacA* | 27 | 100 |
|  | *ugd* | 26 | 96.3 |
| **Phenicol** | *cmlA1* | 2 | 7.4 |
|  | *cmlA6* | 2 | 7.4 |
|  | *floR* | 8 | 29.6 |
| **Phosphonic acid** | *fosA7* | 1 | 3.7 |
|  | *fosA8* | 3 | 11.1 |
|  | *uhpT* | 27 | 100 |
| **Sulfonamide** | *sul2* | 9 | 33.3 |
| **Tetracycline** | *tet(A)* | 9 | 33.3 |
|  | *tet(C)* | 6 | 22.2 |
|  | *tet(M)* | 2 | 7.41 |

**Table S4.** Genomic distribution and IS proximity enrichment of ARG loci (n= 1.148).

| Feature | Chromosome  (n = 729) | Prophage  (n = 285) | Plasmid  (n = 134) | 95% CI | FDR |
| --- | --- | --- | --- | --- | --- |
| Total ARG loci | 729 (63.5 %) | 285 (24.8%) | 134 (11.7%) | – | – |
| IS within 10 kb | 86 (11.8%) | 39 (13.7%) | 57 (42.5%) | 3.59–8.49 | < 6.1 × 10⁻¹⁵ |
| IS within 5 kb | 52 (7.1%) | 29 (10.2%) | 52 (38.8%) | 5.13–13.22 | < 1.5 × 10⁻¹⁸ |
